# Better Prognosis and Survival in Esophageal Cancer Survivors After Comorbid Second Primary Malignancies: A SEER Database-Based Study

**DOI:** 10.3389/fsurg.2022.893429

**Published:** 2022-05-06

**Authors:** Jiayue Ye, Sheng Hu, Wenxiong Zhang, Deyuan Zhang, Yang Zhang, Dongliang Yu, Jinhua Peng, Jianjun Xu, Yiping Wei

**Affiliations:** Department of Thoracic Surgery, The Second Affiliated Hospital of Nanchang University, Nanchang, China

**Keywords:** esophageal cancer survivors, second primary malignant cancer, SEER, survival, prognosis

## Abstract

**Background:**

With the development of surgical techniques and advances in systemic treatments, the survival time of esophageal cancer survivors has increased; however, the chance of developing a second primary malignancy (SPM) has also increased. These patients’ prognosis and treatment plans remain inconclusive.

**Objectives:**

We aimed to evaluate and predict the survival of patients with esophageal cancer with second primary tumors, to provide insights and the latest data on whether to pursue more aggressive treatment.

**Materials and Methods:**

We selected esophageal cancer cases from the latest available data from the SEER database on April 15, 2021. We performed life table analysis, Kaplan–Meier analysis, and univariate and multivariate Cox proportional hazards analysis to assess the patient data. We conducted multiple Cox regression equation analyses under multiple covariate adjustment models, and performed a stratified analysis of multiple Cox regression equation analysis based on different covariates. To describe our study population more simply and clearly, we defined the group of patients with esophageal cancer combined with a second primary malignant tumor (the first of two or more primaries) as the EC-SPM group.

**Results:**

Our analysis of 73,456 patients with esophageal cancer found the median survival time of the EC-SPM group was 47.00 months (95% confidence interval (CI), 43.87–50.13), and the mean survival time was 74.67 months (95% CI, 72.12–77.22). Kaplan–Meier curves of different esophageal cancer survivors showed that the survival of the EC-SPM group was significantly better than that of the other groups (*p* < 0.01). Univariate Cox regression analysis showed that compared with only one malignancy only group, the hazard ratio (HR) of the EC-SPM group was 0.95 (95% CI, 0.92–0.99; *p* < 0.05). In the multivariate Cox regression analysis under different adjustment models, the EC-SPM group had a reduced risk of death compared with the one primary malignancy only group (HR < 1, *p* < 0.05).

**Conclusion:**

Survivors of esophageal cancer with a second primary malignant cancer have a better prognosis, but require more aggressive treatment. This study provided new evidence and new ideas for future research on the pathophysiological mechanism and treatment concepts of esophageal cancer combined with SPM.

## Introduction

Esophageal cancer (EC) is the seventh most common cancer in the world and the sixth leading cause of cancer death (1, 2). In recent years, with the progress of surgical technology and systematic treatment, the survival time of patients with cancer has improved significantly (3). Therefore, the problem of cancer survivors complicated with a second primary malignant tumor (SPM) has become more prominent (4, 5). The treatment plan for patients with esophageal cancer combined with an SPM has not yet been finalized, which poses new challenges for clinicians (6, 7).

SPM refers to tumor occurrence in a single or multiple organs of the same individual, developing after the first primary malignancy, independent of the first primary malignancy, rather than through metastasis or recurrence (8). Mechanistic research into SPM is vague, showing that it might be related to genetics (9), treatment-related exposures (such as radiation therapy) (10), and behavior-related factors (11).

In the past, patients with esophageal cancer with SPM were considered at risk of poor prognosis, and more aggressive treatment might be abandoned as a result. Previous studies had limitations, such as obsolete data and cases that could not represent the esophageal cancer population adequately; therefore, their conclusions were controversial (3, 12–18). Currently, there is no relevant prospective research, and the presence of controversial research makes it difficult for clinicians to guide treatment plans accurately. Surveillance, Epidemiology, and End Results (SEER) is the authoritative source of cancer statistics in the United States. The SEER database released the most recent esophageal cancer follow-up data on April 15, 2021. Therefore, the data sources are very representative. A comprehensive understanding of the prognosis and influencing factors of esophageal cancer with SPM might provide new evidence and support for future research on disease mechanisms and treatment concepts.

Our objective was to further investigate the true survival of patients with esophageal cancer combined with SPM based on the latest data, providing an update on the evidence that such patients should be treated more aggressively.

## Materials and Methods

### Data Sources

Data for our study were obtained from the SEER database (https://www.cancer.gov) on April 15, 2021, and we included data from 18 US states from 2000 to 2018 (including San Francisco Oakland standard metropolitan statistical area (SMSA), Connecticut, Detroit (Metropolitan), Hawaii, Iowa, New Mexico, Seattle (Puget Sound), Utah, Atlanta (Metropolitan), San Jose Monterey, Los Angeles, Alaska Natives, rural Georgia) California, Kentucky, Louisiana, New Jersey, and greater Georgia) comprising records of patients with newly diagnosed esophageal cancer. All patients with esophageal cancer were included in our study. Data for the study’s exposure variables and dependent variables were complete, with no missing values. Missing values for some covariates were imputed as an independent group and named “unknown”. Our study covered 27.8% of the US population (based on the 2010 Census). We selected 13 entries including ID, survival months (the median and mean survival time of patients with esophageal cancer and a second primary malignancy was calculated from the date of diagnosis of esophageal cancer), status, year of diagnosis, sex, age, ethnicity (White, Black, Asian, Pacific Islander and Native American/Native Alaskan), International Classification of Diseases for Oncology, 3rd Edition (ICD-O-3) histological type, primary site, grade (through 2017), summary stage 2000 (1998–2017), median household income inflation-adjusted to 2019, regional nodes positive (1988+), and a total number of in situ/malignant tumors for the patient. Institutional review board approval was not necessary because the SEER database is publicly available.

### Data Grouping

Individual entries were integrated and grouped (Supplementary Tables S1, S2). To conduct the study more clearly and simply, we defined the group having the 1st of two or more primaries in our study as the EC-SPM group. There were no missing values for age, primary site, and histological type (ICD-O-3) and a small number of missing values for other variables; however, these were all rank or quantile variables given a fill-in using the median or mode.

### Data Processing and Statistical Analysis

We use frequency function statistics, and SPSS v. 24 (IBM Corp., Armonk, NY, USA) for the statistical analysis. We used GraphPad Prism 8 (GraphPad Inc., La Jolla, CA, USA) to plot the trend of median survival time in the different subgroups. Data were analyzed using statistical packages R version 3.6.3 (R Foundation, http://www.r-project.org) and Empower Stats (www.empowerstats.net, X&Y solutions Inc., Boston, Massachusetts). *P* value <0.05 was considered statistically significant. Life table, Kaplan–Meier, and univariate and multivariate Cox proportional hazards analyses were used to study the differences in prognosis and we performed overall analysis and stratified analysis using multivariate Cox regression with multiple adjustment models using sequence number as the exposure variable. Model I was not adjusted. Model II was adjusted for age, sex, and ethnicity. Model III was adjusted according to age, sex, ethnicity, histological type, summary stage, regional nodes positive, primary site, and household income. Log rank (Mantel-Cox), Breslow (generalized Wilcoxon), and Tarone–Ware tests were used to compare the distribution of survival data between the groups.

## Results

There were 73,456 patients diagnosed with esophageal cancer entered into the SEER database from 2000 to 2017, of which 77.31% were male, 69.36% were under 75 years old, 46.24% were in the esophageal squamous-cell carcinoma (ESCC) group, 20.08% were in the localized group, the lymph nodes not examined group account for 77.67%, the lower third of esophagus group accounted for 56.50%, and the income group less than $75,000 accounted for 69.99%. The remaining baseline data for the populations are presented in Table 1. The comparison of median survival time and the growth rate of each group is shown in Supplementary Figure S1.

**Table 1:** Baseline characteristics of participants (*N* = 73,456).

Sequence number	*N* (%)	One primary only	1st of 2 or more primaries	2nd of 2 or more primaries	3 or more primaries	*p*-value
**Sex (%)**						<0.01
Female	16671 (22.69%)	21.8	21.2	24.6	32.8	
Male	56785 (77.31%)	78.2	78.8	75.4	67.2	
**Age (%)**						<0.01
≤74 years	50951 (69.36%)	73.0	77.0	56.3	47.3	
75+ years	22505 (30.64%)	27.0	23.0	43.7	52.7	
**Race (%)**						<0.05
White and other races	65303 (88.90%)	88.8	88.0	89.0	90.5	
Black	8153 (11.10%)	11.2	12.0	11.0	9.5	
**Histologic type (%)**						<0.01
Adenocarcinomas	39491 (53.76%)	55.3	54.5	49.9	41.3	
Squamous cell neoplasia and other types	33965 (46.24%)	44.7	45.5	50.1	58.7	
**Summary stage (%)**						<0.01
Localized	14750 (20.08%)	17.7	34.6	24.2	26.4	
Regional	34937 (47.56%)	47.1	48.2	48.7	51.3	
Distant	23769 (32.36%)	35.2	17.2	27.1	22.2	
**Regional nodes positive (%)**						<0.01
Lymph nodes not examined	57053 (77.67%)	77.3	66.4	81.1	85.5	
Lymph nodes were negative	8871 (12.08%)	11.9	22.3	10.6	8.4	
Lymph nodes were positive	7532 (10.25%)	10.9	11.3	8.3	6.1	
**Primary site (%)**						<0.01
Lower third of esophagus	41502 (56.50%)	57.8	58.7	52.4	46.2	
Other sites	31954 (43.50%)	42.2	41.3	47.6	53.8	
**Income (%)**						<0.01
<$75,000	51412 (69.99%)	70.5	69.5	68.6	67.2	
$75,000+	22044 (30.01%)	29.5	30.5	31.4	32.8	
**Sequence number**						<0.01
One primary only	54219 (73.81%)	100	0	0	0	
1st of 2 or more primaries	3923 (5.34%)	0	100	0	0	
2nd of 2 or more primaries	12394 (16.87%)	0	0	100	0	
3 or more primaries	2920 (3.98%)	0	0	0	100	
**Status (%)**						<0.01
Alive	12222 (16.64%)	16.3	28.4	15.0	14.2	
Dead	61234 (83.36%)	83.7	71.6	85.0	85.8	

*Note: (a) Other ethnicities included Asian, Pacific Islander and Native American/Native Alaskan.*

*(b) Other types included the histological types of esophageal cancer except for adenocarcinoma and squamous cell carcinoma.*

*(c) Others included C15.0-Cervical esophagus, C15.1-Thoracic esophagus, C15.2-Abdominal esophagus, C15.3-Upper third of esophagus, C15.4-Middle third of esophagus, C15.8-Overlapping lesion of esophagus, and C15.9-Esophagus, NOS.*

### Better Survival and Prognosis in Patients With Esophageal Cancer Combined With SPM

#### The Survival Advantage of Patients with Esophageal Cancer Combined With SPM

**Figure 1:**
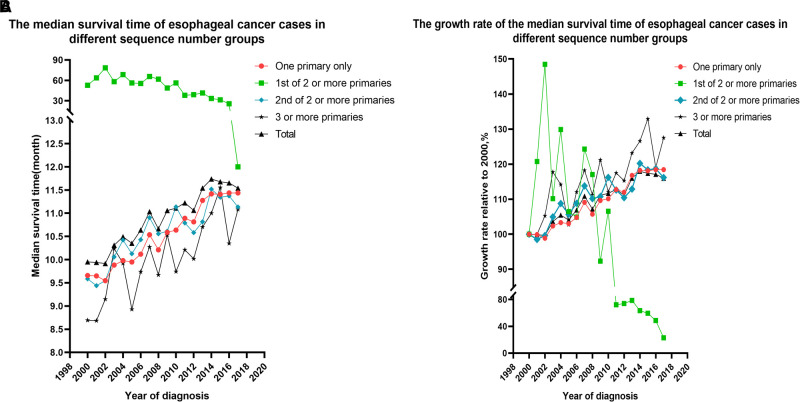
Changes to the median survival time and the growth rate of median survival time in patients with esophageal cancer from 2000 to 2017, according to the sequence number. (**A**) Changes in median survival time. (**B**) Changes in growth rate.

The median survival time of the 73,456 patients was 10.00 months (95% confidence interval (CI), 9.87–10.14), the mean survival time was 33.44 months (95% CI, 32.95–33.93), and the five-year survival rate was 14% (*p* < 0.01). The median survival time of the EC-SPM group was 47.00 months (95% CI, 43.87–50.13), the mean survival time was 74.67 months (95% CI, 72.12–77.22), and the five-year survival rate was 39% (*p* < 0.01). The median survival time of the one primary malignancy only group was 9.00 months (95% CI, 8.86–9.14), the mean survival time was 32.16 months (95% CI, 31.58–32.74), and the five-year survival rate was 13% (*p* < 0.01). The median survival time of the 2nd of two or more primaries group was 9.00 months (95% CI, 8.69–9.32), the mean survival time was 27.94 months (95% CI, 26.95–28.93), and the five-year survival rate was 12% (*p* < 0.01). The median survival time of the 3rd of three or more primaries group was 8.00 months (95% CI, 7.42–8.58), the mean survival time was 23.18 months (95% CI, 21.44–24.93), and the five-year survival rate was 9% (*p* < 0.01) (Table 2 and Supplementary Data Sheet S1). The overall median survival time growth rate was 15.98%, the median survival time growth rate was 18.43% in the one primary only group, and the median survival time growth rate was decreased in the EC-SPM group (Figure 1 and Supplementary Data Sheet S2).

**Table 2:** The 3-year and 5-year survival rates of patients with esophageal cancer; Median and mean survival time of patients with esophageal cancer.

	Percentage of total patients (%)	3-year survival rate (%)	Probability density	5-year survival rate (%)	Probability density	10-year survival rate (%)	Probability density	Median survival time (months)	Standard Error	95.0%, CI		Mean survival time (months)	Standard Error	95.0%, CI	
										Lower	Upper			Lower	Upper
**Total**	100.00	18.00	<0.01	14.00	<0.01	9.00	<0.01	10.00	0.07	9.87	10.14	33.44	0.25	32.95	33.93
**Sex**															
Female	22.70	18.00	<0.01	14.00	<0.01	9.00	<0.01	9.00	0.14	8.72	9.28	33.26	0.52	32.25	34.27
Male	77.30	18.00	<0.01	14.00	<0.01	9.00	<0.01	10.00	0.08	9.85	10.15	33.48	0.28	32.93	34.04
**Age**															
<75 years	69.40	24.00	<0.01	18.00	<0.01	12.00	<0.01	11.00	0.10	10.81	11.19	39.84	0.33	39.18	40.49
75+ years	30.60	13.00	<0.01	8.00	<0.01	3.00	<0.01	6.00	0.09	5.83	6.17	18.95	0.26	18.44	19.46
**Race**															
White and other races (a)	88.90	21.00	<0.01	16.00	<0.01	10.00	<0.01	10.00	0.07	9.86	10.14	34.70	0.27	34.17	35.23
Black	11.10	14.00	<0.01	10.00	<0.01	5.00	<0.01	7.00	0.15	6.70	7.30	23.56	0.58	22.43	24.69
**Histologic Type (ICD-O-3)**															
Adenocarcinomas	53.80	23.00	<0.01	17.00	<0.01	11.00	<0.01	11.00	0.11	10.78	11.22	38.78	0.38	38.05	39.52
Squamous cell neoplasia and other types (b)	46.20	17.00	<0.01	12.00	<0.01	7.00	<0.01	8.00	0.08	7.84	8.16	27.35	0.31	26.74	27.96
**Sequence number**															
One primary only	73.80	16.00	<0.01	13.00	<0.01	9.00	<0.01	9.00	0.07	8.86	9.14	32.16	0.29	31.58	32.74
1st of 2 or more primaries	5.30	50.00	<0.01	39.00	<0.01	22.00	<0.01	47.00	1.60	43.87	50.13	74.67	1.30	72.12	77.22
2nd of 2 or more primaries	16.90	16.00	<0.01	12.00	<0.01	6.00	<0.01	9.00	0.16	8.69	9.32	27.94	0.51	26.95	28.93
3 or more primaries	4.00	14.00	<0.01	9.00	<0.01	4.00	<0.01	8.00	0.30	7.42	8.58	23.18	0.89	21.44	24.93
**Summary stage**															
Localized	20.10	40.00	<0.01	32.00	<0.01	21.00	<0.01	23.00	0.50	22.03	23.97	61.16	0.70	59.79	62.54
Regional	47.60	22.00	<0.01	16.00	<0.01	9.00	<0.01	12.00	0.12	11.77	12.23	35.08	0.38	34.34	35.82
Distant	32.40	6.00	<0.01	4.00	<0.01	2.00	<0.01	5.00	0.06	4.89	5.11	13.43	0.23	12.98	13.88
**Regional nodes positive**															
Lymph nodes not examined	77.70	14.00	<0.01	10.00	<0.01	5.00	<0.01	7.00	0.06	6.89	7.11	23.28	0.22	22.84	23.71
Lymph nodes were negative	12.10	58.00	<0.01	48.00	<0.01	33.00	<0.01	56.00	1.67	52.73	59.27	91.21	1.07	89.12	93.30
Lymph nodes were positive	10.20	24.00	<0.01	16.00	<0.01	10.00	<0.01	16.00	0.28	15.46	16.54	39.56	0.80	37.99	41.13
**Primary Site**															
C15.5-Lower third of esophagus	56.50	23.00	<0.01	17.00	<0.01	11.00	<0.01	11.00	0.10	10.81	11.19	37.94	0.36	37.24	38.65
Other sites (c)	43.50	17.00	<0.01	12.00	<0.01	7.00	<0.01	8.00	0.09	7.83	8.17	27.51	0.33	26.87	28.16
**Income**															
< $75,000	70.00	20.00	<0.01	15.00	<0.01	9.00	<0.01	9.00	0.08	8.85	9.15	32.22	0.29	31.65	32.79
$75,000+	30.00	22.00	<0.01	16.00	<0.01	10.00	<0.01	11.00	0.14	10.74	11.26	36.26	0.48	35.32	37.19

*Note: (a) Other ethnicities included Asian, Pacific Islander and Native American/Native Alaskan.*

*(b) Other types included the histological types of esophageal cancer except for adenocarcinoma and squamous cell carcinoma.*

*(c) Others included C15.0-Cervical esophagus, C15.1-Thoracic esophagus, C15.2-Abdominal esophagus, C15.3-Upper third of esophagus, C15.4-Middle third of esophagus, C15.8-Overlapping lesion of esophagus and C15.9-Esophagus, NOS.*

#### Kaplan–Meier Curves for Survival Advantage of Patients With Esophageal Cancer Combined With SPM

**Figure 2:**
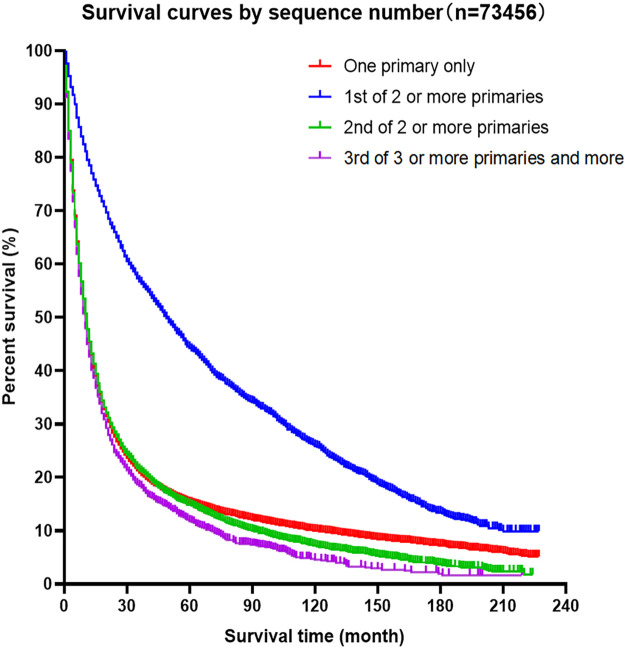
Kaplan–Meier survival curves for patients with esophageal cancer, according to sequence number.

**Figure 3:**
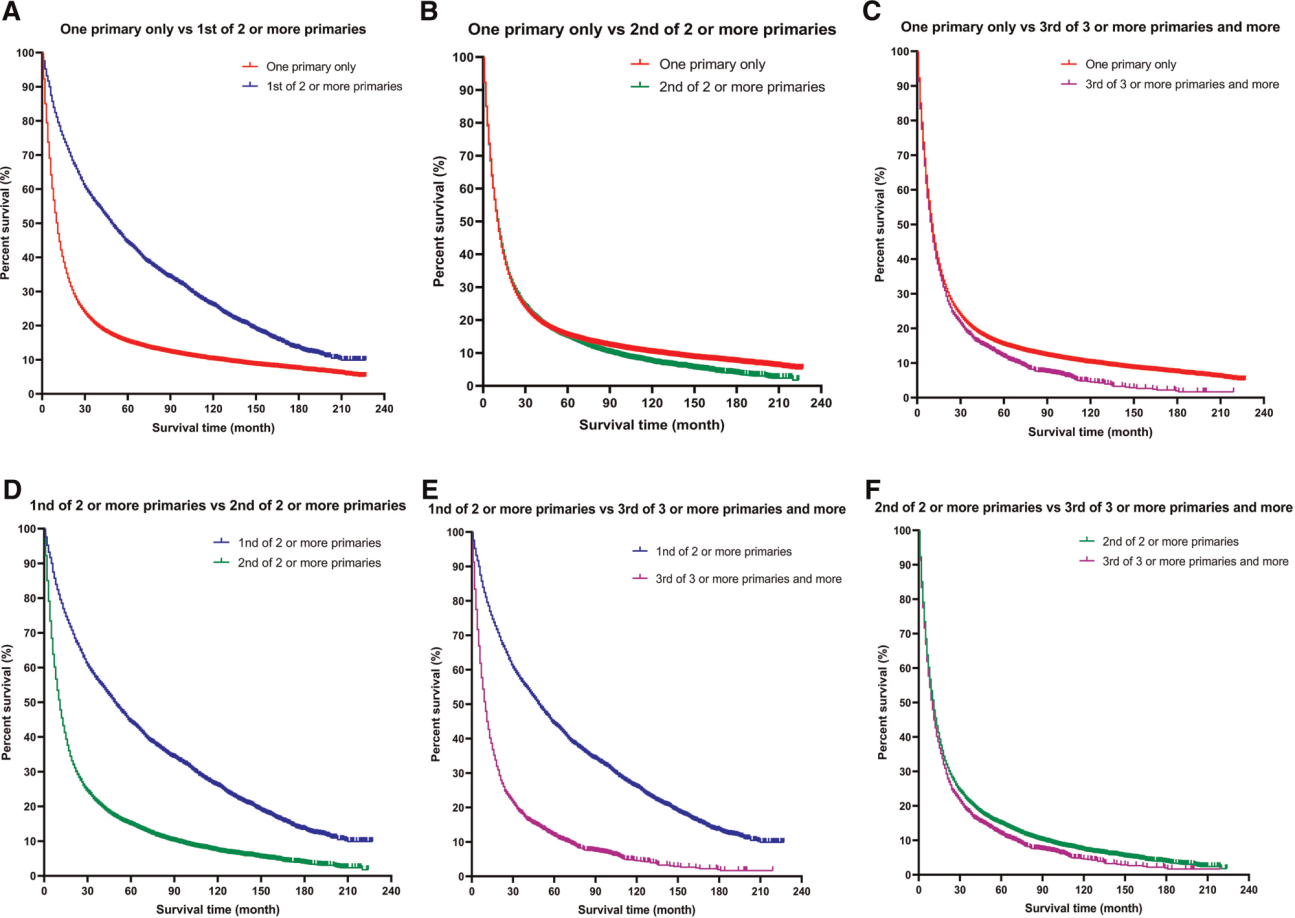
A more detailed classification based on the order of diagnosis of esophageal cancer among multiple primary cancers. (**A**) One primary only vs. 1st of two or more primaries. (**B**) One primary only vs. 2nd of two or more primaries. (**C**) One primary only vs. 3rd of three or more primaries. (**D**) 1st of two or more primaries vs. 2nd of two or more primaries. (**E**) 1st of two or more primaries vs. 3rd of three or more primaries. (**F**) 2nd of two or more primaries vs. 3rd of three or more primaries.

Kaplan–Meier curves of the different groups of esophageal cancer survivors showed that the survival of the EC-SPM group was significantly better than that of the other groups (*p* < 0.01; Figure 2). The survival rate of the one primary malignancy only group was higher than that of 3rd of three or more primaries group (*p* < 0.05); and the survival rate of the 2nd of three or more primaries group was higher than that of the 3rd of three or more primaries group (*p* < 0.05; Figure 3 and Table 3). The Log-rank (Mantel–Cox) test, Breslow (generalized Wilcoxon) test, and Tarone–Ware test were used to indicate significant chi squared and *p*-values for survival differences in the between group comparisons (Table 3).

**Table 3:** Overall comparison and pairwise comparison of each group in Kaplan-Meier survival analysis.

Comparison type	Comparative factor	Log Rank (Mantel-Cox)		Breslow (Generalized Wilcoxon)		Tarone-Ware	
		Chi square	Significance	Chi square	Significance	Chi square	Significance
**Overall comparison**	Age	2450.65	<0.01	2391.85	<0.01	2429.29	<0.01
	Race	359.84	<0.01	304.18	<0.01	343.16	<0.01
	Histologic type (ICD-O-3)	860.26	<0.01	930.70	<0.01	932.94	<0.01
	Sequence number	1758.49	<0.01	1929.31	<0.01	2080.70	<0.01
	Summary stage	9368.91	<0.01	7634.22	<0.01	8771.06	<0.01
	Regional nodes positive	8002.74	<0.01	8010.97	<0.01	8521.65	<0.01
	Primary Site	725.20	<0.01	851.60	<0.01	816.19	<0.01
	Income	109.89	<0.01	136.23	<0.01	131.54	<0.01
**Pairwise comparison**	**Age**						
	75 + years vs. <75 years	2450.65	<0.01	2391.85	<0.01	2429.30	<0.01
	**Race**						
	Black vs. White and other races (a)	359.84	<0.01	304.18	<0.01	343.16	<0.01
	**Histologic type (ICD-O-3)**						
	Squamous cell neoplasia and other types (b) vs. Adenocarcinomas	860.26	<0.01	930.70	<0.01	932.94	<0.01
	**Sequence number**						
	1st of 2 or more primaries vs. One primary only, 2nd of 2 or more primaries and 3 or more primaries	1758.49	<0.01	1929.31	<0.01	2080.70	<0.01
	One primary only vs. 1st of 2 or more primaries	1641.27	<0.01	1888.85	<0.01	2005.1	<0.01
	One primary only vs. 2nd of 2 or more primaries	4.18	0.04	0.01	0.94	0.09	0.76
	One primary only vs. 3 or more primaries	22.82	<0.01	8.75	<0.01	12.46	<0.01
	1st of 2 or more primaries vs. 2nd of 2 or more primaries	1716.00	<0.01	1717.49	<0.01	1843.55	<0.01
	1st of 2 or more primaries vs. 3 or more primaries	1431.69	<0.01	1406.65	<0.01	1470.40	<0.01
	2nd of 2 or more primaries vs. 3 or more primaries	15.16	<0.01	7.761	<0.01	10.48	<0.01
	**Summary stage**						
	Regional vs. Localized	1491.63	<0.01	1119.51	<0.01	1369.08	<0.01
	Distant vs. Localized	7900.62	<0.01	5997.76	<0.01	7111.73	<0.01
	Distant vs. Regional	4756.79	<0.01	4067.00	<0.01	4589.46	<0.01
	**Regional nodes positive**						
	Lymph nodes were negative vs. Lymph nodes not examined	7162.58	<0.01	6529.65	<0.01	7278.61	<0.01
	Lymph nodes were positive vs. Lymph nodes not examined	1178.81	<0.01	1819.85	<0.01	1628.48	<0.01
	Lymph nodes were positive vs. Lymph nodes were negative	2031.43	<0.01	1,870.28	<0.01	2,041.87	<0.01
	**Primary Site**						
	Other sites (c) vs. C15.5-Lower third of esophagus	725.20	<0.01	851.60	<0.01	816.19	<0.01
	**Income**						
	$75,000+ vs. <$75,000	109.89	<0.01	136.23	<0.01	131.54	<0.01

*Note: (a) Other ethnicities included Asian, Pacific Islander and Native American/Native Alaskan.*

*(b) Other types included the histological types of esophageal cancer except for adenocarcinoma and squamous cell carcinoma.*

*(c) Others included C15.0-Cervical esophagus, C15.1-Thoracic esophagus, C15.2-Abdominal esophagus, C15.3-Upper third of esophagus, C15.4-Middle third of esophagus, C15.8-Overlapping lesion of esophagus and C15.9-Esophagus, NOS.*

#### Univariate and Multivariate Cox Regression Analysis of Survival Advantage in Patients with Esophageal Cancer with SPM

Univariate Cox regression analysis revealed a 5.00% reduction in the risk of death in the EC-SPM group compared with that in the reference group (95% CI, 0.92–0.99; *p* < 0.05) (Table 4). Using sequence number as the exposure variable, survival time as the time variable, status as the outcome variable, and one primary only group as the reference group, Cox multiple regression equation analysis was performed in different models of adjustment (total analysis and stratified analysis) (Table 5). The results of the overall analysis showed that under Model I, the risk of death in the EC-SPM group was 53% lower than that in the reference group (95% CI, 0.45–0.49; *p* < 0.01). After adjustment in model II, the risk of death in the EC-SPM group was reduced by 53% (95% CI, 0.45–0.48; *p* < 0.01). After adjustment for Model III, the risk of death in the EC-SPM group was reduced by 49% (95% CI, 0.49–0.53; *p* < 0.01). When adjusted according to model III, the risk of death in the EC-SPM group was reduced in the subgroups of age, sex, ethnicity, histological type, summary stage, primary site, lymph node positive, and household income (hazard ratio (HR) <1, *p* < 0.05). The more detailed results, the 95% CI, and *p*-values are shown in Table 5.

**Table 4:** Univariate Cox proportional hazards analysis of esophageal cancer based on the SEER database.

Sub-group	Univariate analysis	*p*-value
	HR (95%CI)	
**Sequence number**		
One primary only	Reference (1)	
1st of 2 or more primaries	0.95 (0.92-0.99)	<0.01
2nd of 2 or more primaries	0.98 (0.96-1.00)	<0.01
3 or more primaries	0.93 (0.89-0.96)	<0.01
**Age**		
≤74 years	Reference (1)	
75+ years	1.16 (1.15-1.18)	<0.01
**Sex**		
Female	Reference (1)	
Male	0.96 (0.94-0.98)	<0.01
**Race**		
White and other races(a)	Reference (1)	
Black	1.22 (1.19-1.25)	<0.01
**Histologic type**		
Adenocarcinomas	Reference (1)	
Squamous cell neoplasia and other types (b)	1.26 (1.24-1.28)	<0.01
**Summary stage**		
Localized	Reference (1)	
Regional	0.77 (0.75-0.78)	<0.01
Distant	1.14 (1.12-1.17)	<0.01
**Regional nodes positive**		
Lymph nodes were negative	Reference (1)	
Lymph nodes were positive	2.07 (2.00-2.15)	<0.01
Lymph nodes not examined	3.27 (3.18-3.37)	<0.01
**Primary Site**		
Lower third of esophagus	Reference (1)	
other sites(c)	1.13 (1.11-1.15)	<0.01
**Household income**		
<$75,000	Reference (1)	
$75,000+	0.97 (0.96-0.99)	<0.01

*Note: (a) Other ethnicities included Asian, Pacific Islander and Native American/Native Alaskan.*

*(b) Other types included the histological types of esophageal cancer except for adenocarcinoma and squamous cell carcinoma.*

*(c) Others included C15.0-Cervical esophagus, C15.1-Thoracic esophagus, C15.2-Abdominal esophagus, C15.3-Upper third of esophagus, C15.4-Middle third of esophagus, C15.8-Overlapping lesion of esophagus and C15.9-Esophagus, NOS.*

**Table 5:** Cox multiple regression equation analysis in different models of adjustment (total analysis and stratified analysis).

Outcome	Model I	Model II	Model III
	HR (95%CI) *p*-value	HR (95%CI) *p*-value	HR (95%CI) *p*-value
**Total**			
One primary only	Reference (1)	Reference (1)	Reference (1)
1st of 2 or more primaries	0.47 (0.45, 0.49) <0.01	0.47 (0.45, 0.48) <0.01	0.51 (0.49, 0.53) <0.01
2nd of 2 or more primaries	1.02 (1.00, 1.05) <0.01	0.95 (0.93, 0.97) <0.01	1.00 (0.96 1.00) 0.04
3 or more primaries	1.11 (1.07, 1.16) <0.01	1.00 (1.00, 1.04) 0.92	1.03 (0.99, 1.07) 0.17
**Age**			
≤74 years			
One primary only	Reference (1)	Reference (1)	Reference (1)
1st of 2 or more primaries	0.46 (0.44, 0.49) <0.01	0.46 (0.44, 0.48) <0.01	0.50 (0.48, 0.52) <0.01
2nd of 2 or more primaries	0.98 (0.95, 1.01) 0.16	0.98 (0.95, 1.01) 0.12	1.01 (0.98, 1.04) 0.61
3 or more primaries	1.05 (0.99, 1.11) 0.14	1.05 (0.99, 1.12) 0.09	1.08 (1.02, 1.15) 0.01
75+ years			
One primary only	Reference (1)	Reference (1)	Reference (1)
1st of 2 or more primaries	0.48 (0.45, 0.52) <0.01	0.48 (0.45, 0.52) <0.01	0.53 (0.49, 0.57) <0.01
2nd of 2 or more primaries	0.92 (0.89, 0.95) <0.01	0.92 (0.89, 0.95) <0.01	0.95 (0.92, 0.98) <0.01
3 or more primaries	0.96 (0.91, 1.01) 0.13	0.96 (0.91, 1.01) 0.14	0.99 (0.93, 1.05) 0.69
**Sex**			
Female			
One primary only	Reference (1)	Reference (1)	Reference (1)
1st of 2 or more primaries	0.46 (0.42, 0.50) <0.01	0.47 (0.43, 0.51) <0.01	0.50 (0.46, 0.54) <0.01
2nd of 2 or more primaries	0.99 (0.95, 1.03) 0.66	0.96 (0.92, 1.00) <0.05	0.99 (0.95, 1.04) 0.67
3 or more primaries	1.01 (0.94, 1.09) 0.71	0.96 (0.89, 1.03) 0.23	1.01 (0.94, 1.09) 0.82
Male			
One primary only	Reference (1)	Reference (1)	Reference (1)
1st of 2 or more primaries	0.47 (0.45, 0.49) <0.01	0.47 (0.45, 0.49) <0.01	0.52 (0.49, 0.54) <0.01
2nd of 2 or more primaries	1.03 (1.01, 1.06) <0.01	0.95 (0.93, 0.98) <0.01	0.98 (0.96, 1.00) 0.10
3 or more primaries	1.16 (1.10, 1.21) <0.01	1.02 (0.97, 1.08) 0.37	1.05 (1.00, 1.10) 0.08
**Race**			
White and other races (a)			
One primary only	Reference (1)	Reference (1)	Reference (1)
1st of 2 or more primaries	0.47 (0.45 0.49) <0.01	0.47 (0.45, 0.49) <0.01	0.52 (0.50, 0.54) <0.01
2nd of 2 or more primaries	1.03 (1.01, 1.06) <0.01	0.95 (0.93, 0.98) <0.01	0.98 (0.96, 1.01) 0.15
3 or more primaries	1.14 (1.09, 1.19) <0.01	1.01 (0.97, 1.05) 0.66	1.04 (0.99, 1.08) 0.10
Black			
One primary only	Reference (1)	Reference (1)	Reference (1)
1st of 2 or more primaries	0.45 (0.40, 0.50) <0.01	0.45 (0.41, 0.50) <0.01	0.47 (0.42, 0.52) <0.01
2nd of 2 or more primaries	0.94 (0.89, 1.00) 0.06	0.91 (0.86, 0.97) <0.01	0.93 (0.88, 0.99) 0.03
3 or more primaries	0.91 (0.80, 1.03) 0.15	0.88 (0.78, 1.00) 0.06	0.96 (0.84, 1.09) 0.51
**Histologic type**			
Adenocarcinomas			
One primary only	Reference (1)	Reference (1)	Reference (1)
1st of 2 or more primaries	0.47 (0.45, 0.50) <0.01	0.47 (0.44, 0.49) <0.01	0.55 (0.52, 0.58) <0.01
2nd of 2 or more primaries	1.05 (1.02, 1.08) <0.01	0.94 (0.91, 0.97) <0.01	1.00 (0.97, 1.03) 0.84
3 or more primaries	1.15 (1.08, 1.23) <0.01	0.98 (0.92, 1.05) 0.58	1.04 (0.97, 1.11) 0.24
Squamous cell neoplasia and other types (b)			
One primary only	Reference (1)	Reference (1)	Reference (1)
1st of 2 or more primaries	0.45 (0.43, 0.48) <0.01	0.46 (0.43, 0.48) <0.01	0.48 (0.46, 0.51) <0.01
2nd of 2 or more primaries	0.97 (0.94, 1.00) <0.05	0.94 (0.91, 0.97) <0.01	0.96 (0.93, 0.99) <0.01
3 or more primaries	1.01 (0.96, 1.06) 0.72	0.97 (0.92, 1.02) 0.25	1.02 (0.96, 1.07) 0.58
**Summary stage**			
Localized			
One primary only	Reference (1)	Reference (1)	Reference (1)
1st of 2 or more primaries	0.60 (0.56, 0.64) <0.01	0.60 (0.56, 0.64) <0.01	0.59 (0.549, 0.631) <0.01
2nd of 2 or more primaries	1.20 (1.15, 1.26) <0.01	1.07 (1.02, 1.12) <0.01	1.02 (0.970, 1.065) 0.49
3 or more primaries	1.48 (1.36, 1.60) <0.01	1.20 (1.11, 1.31) <0.01	1.11 (1.020, 1.200) <0.05
Regional			
One primary only	Reference (1)	Reference (1)	Reference (1)
1st of 2 or more primaries	0.51 (0.48, 0.54) <0.01	0.52 (0.488, 0.55) <0.01	0.51 (0.49, 0.54) <0.01
2nd of 2 or more primaries	1.11 (1.07, 1.14) <0.01	1.01 (0.98, 1.05) 0.44	0.98 (0.95, 1.01) 0.13
3 or more primaries	1.22 (1.15, 1.30) <0.01	1.08 (1.02, 1.14) <0.05	1.02 (0.96, 1.08) 0.61
Distant			
One primary only	Reference (1)	Reference (1)	Reference (1)
1st of 2 or more primaries	0.45 (0.42, 0.49) <0.01	0.45 (0.42, 0.49) <0.01	0.45 (0.41, 0.49) <0.01
2nd of 2 or more primaries	1.05 (1.01, 1.09) <0.05	0.98 (0.94, 1.02) 0.31	0.97 (0.94, 1.01) 0.17
3 or more primaries	1.09 (1.01, 1.18) <0.05	1.01 (0.93, 1.09) 0.90	1.00 (0.92, 1.08) 0.96
**Regional nodes positive**			
Lymph nodes not examined			
One primary only	Reference (1)	Reference (1)	Reference (1)
1st of 2 or more primaries	0.45 (0.43, 0.47) <0.01	0.45 (0.43, 0.47) <0.01	0.49 (0.47, 0.52) <0.01
2nd of 2 or more primaries	0.95 (0.93, 0.97) <0.01	0.91 (0.89, 0.93) <0.01	0.96 (0.94 0.98) <0.01
3 or more primaries	1.00 (0.95, 1.04) 0.82	0.94 (0.90, 0.98) <0.01	1.02 (0.97, 1.06) 0.48
Lymph nodes were negative			
One primary only	Reference (1)	Reference (1)	Reference (1)
1st of 2 or more primaries	0.67 (0.61, 0.74) <0.01	0.66 (0.60, 0.73) <0.01	0.69 (0.62, 0.6) <0.01
2nd of 2 or more primaries	1.30 (1.20, 1.40) <0.01	1.19 (1.10, 1.29) <0.01	1.22 (1.13, 1.31) <0.01
3 or more primaries	1.48 (1.26, 1.72) <0.01	1.33 (1.14, 1.56) <0.01	1.29 (1.11, 1.51) <0.01
Lymph nodes were positive			
One primary only	Reference (1)	Reference (1)	Reference (1)
1st of 2 or more primaries	0.50 (0.45, 0.56) <0.01	0.49 (0.440, 0.55) <0.01	0.49 (0.44, 0.55) <0.01
2nd of 2 or more primaries	1.05 (0.97, 1.13) 0.24	0.99 (0.92, 1.06) 0.71	0.98 (0.91, 1.06) 0.65
3 or more primaries	1.03 (0.87, 1.22) 0.71	0.94 (0.79, 1.11) 0.44	0.89 (0.75, 1.05) 0.17
**Primary site**			
Lower third of esophagus			
One primary only	Reference (1)	Reference (1)	Reference (1)
1st of 2 or more primaries	0.48 (0.45, 0.50) <0.01	0.47 (0.45, 0.50) <0.01	0.54 (0.51, 0.57) <0.01
2nd of 2 or more primaries	1.05 (1.02, 1.09) <0.01	0.95 (0.92, 0.98) <0.01	1.01 (0.98, 1.04) 0.47
3 or more primaries	1.16 (1.09, 1.23) <0.01	1.01 (0.95, 1.08) 0.69	1.10 (1.03, 1.17) <0.01
Other sites (c)			
One primary only	Reference (1)	Reference (1)	Reference (1)
1st of 2 or more primaries	0.45 (0.42, 0.47) <0.01	0.45 (0.42, 0.48) <0.01	0.48 (0.45, 0.51) <0.01
2nd of 2 or more primaries	0.96 (0.93, 0.99) <0.01	0.93 (0.90, 0.95) <0.01	0.97 (0.94, 1.00) 0.08
3 or more primaries	1.01 (0.95, 1.06) 0.81	0.95 (0.90, 1.01) 0.09	1.02 (0.96, 1.07) 0.61
**Household income**			
≤$75,000			
One primary only	Reference (1)	Reference (1)	Reference (1)
1st of 2 or more primaries	0.47 (0.45, 0.49) <0.01	0.47 (0.45, 0.49) <0.01	0.52 (0.49, 0.54) <0.01
2nd of 2 or more primaries	1.02 (1.00, 1.05) 0.07	0.96 (0.93, 0.98) <0.01	0.98 (0.96, 1.01) 0.12
3 or more primaries	1.12 (1.07, 1.17) <0.01	1.01 (0.96, 1.06) 0.74	1.03 (0.98, 1.08) 0.26
$75,000+			
One primary only	Reference (1)	Reference (1)	Reference (1)
1st of 2 or more primaries	0.46 (0.43, 0.50) <0.01	0.47 (0.44, 0.50) <0.01	0.51 (0.47, 0.54) <0.01
2nd of 2 or more primaries	1.03 (0.99, 1.07) 0.12	0.94 (0.91, 0.98) <0.01	0.97 (0.94, 1.01) 0.17
3 or more primaries	1.10 (1.03, 1.19) <0.01	0.98 (0.91, 1.06) 0.62	1.03 (0.96, 1.11) 0.42

*Note: (a) Other ethnicities included Asian, Pacific Islander and Native American/Native Alaskan. (b) Other types included the histological types of esophageal cancer except for adenocarcinoma and squamous cell carcinoma. (c) Others included C15.0-Cervical esophagus, C15.1-Thoracic esophagus, C15.2-Abdominal esophagus, C15.3-Upper third of esophagus, C15.4-Middle third of esophagus, C15.8-Overlapping lesion of esophagus, and C15.9-Esophagus, NOS. Result variable: Status. Exposure variable: Sequence number. Time variable: Survival months Model I is not adjusted. Model II was adjusted for age, sex, and ethnicity. Model III was adjusted for age, sex, ethnicity, histological type, summary stage, regional nodes positive, primary site, and household income.*

### Variables Influencing the Survival of Patients with Esophageal Cancer

The median survival time and mean survival time of the different groups with different covariates are described in detail in Supplementary Data Sheet S1. The younger than 75 years old group, the non-black group, the adenocarcinomas group, the limited group, the lymph node negative group, and the lower third of the esophageal group had a longer median survival time (Supplementary Figure S2, Data Sheet S1). Kaplan–Meier survival curves of the different covariates showed significant differences in overall survival rates between the different groups, *p* < 0.01 (Supplementary Figure S2 and Table 2).

Univariate Cox regression analysis shows that compared with the reference group, the group younger than 75 years old (HR = 1.16, 95% CI, 1.15–1.18), the black group (HR = 1.22, 95% CI, 1.19–1.25), the non-adenocarcinoma group (HR = 1.26, 95% CI, 1.24–1.28), the distant group (HR = 1.14, 95% CI, 1.12–1.17), the lymph node positive group (HR = 2.07, 95% CI, 2.00–2.15), the lymph node unexamined group (HR = 3.27, 95% CI, 3.18–3.37), and the other site group (HR = 1.13, 95% CI, 1.11–1.15) had higher risk of death (*p* < 0.01). The male group (HR = 0.99, 95% CI, 0.94–0.98), the regional group (HR = 0.77, 95% CI, 0.75–0.78), and the income $75,000 + group (HR = 0.97, 95% CI, 0.96–0.99) had a lower risk of death (*p* < 0.05) (Table 4).

## Discussion

The development of surgical methods and advances in radiotherapy and chemotherapy technology have prolonged the survival time of patients with cancer. Studies have shown that patients with cancer have a higher risk of subsequent cancer than the general population (19–21). With the prolonged survival time of cancer survivors, the incidence of SPM has increased (22–26). Principles of management of multiple primary cancers are distinguished from common metastatic and recurrent cancers, and usually require comprehensive consideration from many aspects (27, 28). Therefore, the prognosis of patients with multiple primary cancers and the choice of treatment represent a new challenge for clinicians (3, 13). Previous studies analyzed the incidence rate (29, 30) of esophageal cancer and the survival rate (15, 16, 31–33) of patients with esophageal cancer. Although some preliminary explorations have been carried out, these studies had a short time span, a low amount of case data, the type of pathology was not described comprehensively, and the study methods were relatively simple. The follow-up data of esophageal cancer from the SEER database were updated in April 2021; therefore, it is necessary to conduct more in-depth studies on esophageal cancer combined with SPM based on the most recent data.

In many cancers, SPMs are considered a risk factor for poor prognosis. Research by Donin et al. showed that 1 out of 12 general cancer survivors suffer from SPM, and for patients with two types of cancer, 13% of patients died from initial cancer, but more than half (55%) died of SPM (22). Van lierde et al. showed that second primary tumors increased mortality significantly in patients with head and neck squamous cell carcinoma (34). Wu et al. showed that the prognosis of patients with SPM with non-small cell lung cancer is poor (35). Several studies have shown that the overall survival rate of patients with primary cancer of grade II or higher might be significantly lower than that of patients with grade I primary cancer I (36–39). However, the above conclusion might not be appropriate in patients with esophageal cancer. Nandy et al. believed that the survival rates of patients with esophageal cancer with or without SPM are similar (26). Some scholars believe that the main determinants of prognosis in patients with esophageal cancer complicated with SPM might be related to patient clinical factors (such as stage), but not the development of SPM. The conclusions of these studies differ from ours. This might reflect differences in research data sources and analysis methods such that the potential differences in the prognosis of the two groups of patients have not been revealed. Duchateau (4) showed that the prognosis of cancer survivors with SPMS is not necessarily very poor, which is similar to the conclusion of the present study. With the prognosis and active treatment of patients with esophageal cancer with SPM receiving increased attention (5), the above-mentioned studies have obvious controversies and limitations (18, 35, 40, 41), and it is difficult to provide convincing, satisfactory, and consistent conclusions to help clinicians diagnose and treat these patients. Therefore, it is very important to conduct more in-depth research based on the updated large sample size of SEER data, the complete pathological types of esophageal cancer, and multiple analysis methods.

Through further analysis, we found that the median survival time of the EC-SPM group was longer. The Kaplan–Meier curve showed that the survival rate of esophageal cancer combined with SPM was higher, and univariate and multivariate Cox regression analysis results showed that the risk of death in the EC-SPM group was lower than that in the one primary malignancy only group. We considered that multiple surgeries, and repeated radiotherapy and chemotherapy might explain the better prognosis of patients with esophageal cancer with SPM compared with those without SPM. During the treatment of secondary cancer, frequent examination, radiotherapy, and chemotherapy might inhibit the recurrence and metastasis of esophageal cancer (13), thereby improving the overall curative effect. Patients with esophageal cancer usually present with an impaired immune ability, including an impaired complement activation pathway (42), while the treatment of second primary cancer might reactivate the immune system and exert antitumor effects (43). This interesting finding provides new insights and evidence for the need for further active treatment for esophageal cancer survivors with SPMs. In addition, our research showed that among cancer survivors, the survival rates of patients whose second primary cancer is esophageal cancer and patients with only esophageal cancer were statistically different. This differed from the results of some previous studies (36–39), and might have been caused by different data sources and statistical methods. However, this study is a retrospective study with a large sample size. In addition, multiple regression equation analysis of the Cox model was performed with multiple different models of variable adjustment, aiming to eliminate the interference of other covariates, which might have made our results more convincing.

Previous studies that carried out analysis of covariate in an identical way to that in the current study, e.g., Schlottmann et al., showed that surgical resection was rarely used in patients with esophageal adenocarcinoma who were aged 70 years or older in the United States (44). Moreover, Ruol et al. stated that old age should not be considered a contraindication for esophageal cancer surgery (45). The failure of older adults with esophageal cancer to receive surgery for their treatment perhaps explains the current finding of lower median survival times and growth rates among patients with esophageal cancer aged 75 years and older in the United States. Mariette et al. showed that one of the most important predictors of survival for patients with esophageal cancer is lymph node metastasis (46, 47). Less than one-third of patients in the United States and less than one-tenth of hospitals have fully checked the condition of the patients’ lymph nodes (48). Our research showed that compared with patients with positive lymph node examinations, patients with esophageal cancer who have not undergone lymph node examination have a shorter median survival time. Therefore, improvement of the policies regarding lymph node examinations might reduce the risk of death for most patients with esophageal cancer.

The limitations of this study included the observation that those patients with positive lymph nodes had a better prognosis than those in the group without examined lymph nodes. This might have been because of the low rate of intraoperative assessment of lymph node status, a conclusion that is not strongly representative. Moreover, this study was a retrospective analysis; therefore, our conclusions need to be further verified by future prospective studies. According to the 2010 census, SEER 18 covers about 27.80% of the U.S. population. If we could obtain the whole esophageal cancer data, not limited to the United States, and include more covariates for analysis, our study will be more convincing. We hope to have more data for further research in the future.

## Conclusion

In conclusion, the overall survival of patients with cancer complicated with SPM is poor. However, the occurrence of the SPM in patients with esophageal cancer is not necessarily a risk factor for poor prognosis. This study provided new evidence and new ideas for future research on the pathophysiological mechanism and treatment concepts of esophageal cancer combined with SPM. These findings might provide valuable insights into aggressive treatment options and ongoing surveillance for SPM in esophageal cancer survivors and could help policymakers to monitor public health issues and implement interventions to reduce mortality from esophageal cancer.

## Contribution to the Field Statement

With the development of surgical techniques and advances in systemic treatments, the survival time of cancer survivors has increased; however, the chance of developing a second primary cancer has also increased. The overall survival rate of cancer survivors with second primary malignancies is poor. However, our study suggests that patients with esophageal cancer combined with second primary malignancies could have a better prognosis, and these patients might require more aggressive treatments. Our results provide new evidence and new ideas for future research on the pathophysiological mechanism and treatment concept of esophageal cancer combined with second primary malignant tumors.

## Data Availability

The original contributions presented in the study are included in the article/Supplementary Material, further inquiries can be directed to the corresponding author/s.
