# Supplementary material for: Better Prognosis and Survival in Esophageal Cancer Survivors After Comorbid Second Primary Malignancies: A SEER Database-Based Study
Source: Front Surg. 2022 May 6;9:893429. doi: 10.3389/fsurg.2022.893429 (PMC9235858; doi:10.3389/fsurg.2022.893429)
Supplement: Supplementary file 3 [file fsurg-09-893429_Table_6_v1.docx]

**Table S1. Histological type ICD-O-3 code and corresponding number of esophageal cancer patients.**

| **Histologic type ICD-O-3** | **Number** | |  | **Histologic type ICD-O-3** | | **Number** | |
| --- | --- | --- | --- | --- | --- | --- | --- |
| **ADENOMIE ADENOCARCINOMI (814-838), Adenoma and adenocarcinomas** | | |  | 8072 | | 465 | |
| 8140 | 38063 | |  | 8073 | 17 | | |
| 8141 | 1 | |  | 8074 | 76 | | |
| 8142 | 2 | |  | 8075 | | 8 | |
| 8143 | | 2 |  | 8076 | | 11 | |
| 8144 | 336 | |  | 8082 | 5 | | |
| 8145 | 100 | |  | 8083 | | 225 | |
| 8170 | 1 | |  | 8084 | | 2 | |
| 8200 | 9 | |  | Total | | 24370 | |
| 8201 | 2 | |  | **Other types** | |  | |
| 8210 | 66 | |  | NEOPLASIE, NAS (800), Neoplasia, NOS | | | |
| 8211 | 43 | |  | 8000 | | 8000 | |
| 8240 | 20 | |  | 8001 | | 8001 | |
| 8244 | 10 | |  | Total | | Total | |
| 8246 | 277 | |  | NEOPLASIE EPITELIALI, NAS (801-804), Epithelial neoplasms, NOS | | | |
| 8247 | 1 | |  | 8012 | | | 58 |
| 8249 | 1 | |  | 8013 | | 54 | |
| 8255 | 379 | |  | 8015 | | 1 | |
| 8260 | 131 | |  | 8020 | | 81 | |
| 8261 | 9 | |  | 8021 | | 20 | |
| 8263 | 15 | |  | 8022 | | 3 | |
| 8310 | | 8 |  | 8030 | | 1 | |
| 8323 | 15 | |  | 8031 | | 1 | |
| Total | 39491 | |  | 8032 | | 26 | |
| **NEOPLASIE A CELLULE SQUAMOSE (805-808), Squamous cell neoplasms** | | |  | 8033 | | 44 | |
| 8050 | 4 | |  | 8041 | | 393 | |
| 8051 | 37 | |  | 8042 | 4 | | |
| 8052 | 12 | |  | 8045 | | 25 | |
| 8070 | 21617 | |  | 8046 | | 165 | |
| 8071 | 1891 | |  | Total | | 3739 | |

**Table S1 Continued.**

| **Histologic type ICD-O-3** | **Number** |  | **Histologic type ICD-O-3** | **Number** |
| --- | --- | --- | --- | --- |
| 8094 | 7 |  | Total | 2 |
| Total | 8 |  | Lipomatous neoplasia |  |
| Papilloma and carcinoma with transitional cells | |  | 8850 | 1 |
| 8123 | 7 |  | 8851 | 5 |
| Mucoepidermoid neoplasia |  |  | 8858 | 1 |
| 8430 | 6 |  | Total | 7 |
| Ductal and lobular neoplasia |  |  | Myomatous neoplasia |  |
| 8500 | 2 |  | 8890 | 25 |
| 8510 | 1 |  | 8891 | 1 |
| 8520 | 1 |  | 8895 | 1 |
| Total | 4 |  | 8901 | 1 |
| Acinous cell neoplasia |  |  | 8912 | 1 |
| 8550 | 1 |  | 8920 | 1 |
| Complex epithelial neoplasia |  |  | Total | 30 |
| 8560 | 509 |  | Complex mixed and stromal neoplasia |  |
| 8562 | 2 |  | 8935 | 1 |
| 8570 | 6 |  | 8936 | 76 |
| 8574 | 78 |  | 8940 | 2 |
| 8575 | 1 |  | 8963 | 5 |
| 8576 | 2 |  | 8980 | 22 |
| Total | 598 |  | 8982 | 2 |
| Paragangliomas and glomus tumors | |  | Total | 108 |
| 8710 | 1 |  | Neoplasia of germinal cells |  |
| Melanoma |  |  | 9064 | 1 |
| 8720 | 40 |  | 9065 | 1 |
| 8742 | 1 |  | 9085 | 1 |
| Total | 41 |  | Total | 3 |
| Tumors of the soft parts and sarcomas, NOS | |  | Tumors of blood vessels |  |
| 8800 | 12 |  | 9120 | 4 |
| 8801 | 4 |  | Miscellaneous bone tumors |  |
| 8803 | 1 |  | 9260 | 2 |
| 8804 | 2 |  | Miscellaneous tumors |  |
| 8805 | 1 |  | 9364 | 3 |
| 8806 | 4 |  | Tumors of the nervous sheathes |  |
| Total | 24 |  | 9540 | 1 |
| Fibromatous neoplasia |  |  | Granular cell tumors and alveolar sarcomas of the milli parts | |
| 8810 | 1 |  |  |  |
| 8811 | 1 |  | 9580 | 2 |
