# Supplementary material for: Better Prognosis and Survival in Esophageal Cancer Survivors After Comorbid Second Primary Malignancies: A SEER Database-Based Study
Source: Front Surg. 2022 May 6;9:893429. doi: 10.3389/fsurg.2022.893429 (PMC9235858; doi:10.3389/fsurg.2022.893429)
Supplement: Supplementary file 4 [file fsurg-09-893429_Table_7_v1.docx]

**Table S2. Detailed grouping of different variables.**

| **Variable** | **Number** | **Group** |
| --- | --- | --- |
| Age | 1 | <75 years |
|  | 2 | 75+ years |
| Race（W、B、AI、API） | 1 | White and other(a） |
|  | 2 | Black |
| Histologic type（ICD-O-3） | 1 | Adenocarcinomas |
|  | 2 | Squamous cell neoplasia and other types (b) |
| Sequence number | 1 | One primary only |
|  | 2 | 1st of 2 or more primaries |
|  | 3 | 2nd of 2 or more primaries |
|  | 4 | 3 or more primaries |
| Summary stage | 1 | Localized |
|  | 2 | Regional |
|  | 3 | Distant |
| Regional nodes positive (1988+) | 1 | Lymph nodes not examined |
|  | 2 | Lymph nodes were negative |
|  | 3 | Lymph nodes were positive |
| Primary site | 1 | C15.5-Lower third of esophagus |
|  | 2 | Other sites (c) |
| Income | 1 | < $75,000 |
|  | 2 | $75,000 |

Note: (a) Other races included Asian, Pacific Islander and Native American/Native Alaskan.

(b) Other types included the histological types of esophageal cancer except for adenocarcinoma and squamous cell carcinoma.

(c) Others included C15.0-Cervical esophagus, C15.1-Thoracic esophagus, C15.2-Abdominal esophagus, C15.3-Upper third of esophagus, C15.4-Middle third of esophagus, C15.8-Overlapping lesion of esophagus and C15.9-Esophagus, NOS.
